# Prenatal Ultrasound Diagnosis of Vasa Previa With Careful Intraoperative Management: A Case Report

**DOI:** 10.7759/cureus.55578

**Published:** 2024-03-05

**Authors:** Sairem M Chanu, Papa Dasari, Chitra T, Bhabani Pegu, Rajalakshmi T

**Affiliations:** 1 Obstetrics and Gynaecology, All India Institute of Medical Sciences, Mangalagiri, IND; 2 Obstetrics and Gynaecology, Jawaharlal Institute of Postgraduate Medical Education and Research (JIPMER), Puducherry, IND; 3 Obstetrics, Jawaharlal Institute of Postgraduate Medical Education and Research (JIPMER), Puducherry, IND

**Keywords:** vasa previa, cesarean section, ultrasound, placenta, prenatal intervention, prenatal diagnosis

## Abstract

Vasa previa is a rare but potentially life-threatening condition to the fetus. Timely antenatal diagnosis and delivery by cesarean section (CS) can lead to a favorable outcome. Here, we report a case of recurrent pregnancy loss (G3A2) with vasa previa, which was diagnosed prenatally by ultrasound. She was admitted at her 31st week with bleeding per vaginum (PV) provisionally diagnosed as antepartum hemorrhage (APH) and managed conservatively as placenta previa. Follow-up ultrasonography (USG) revealed vasa previa at 33 weeks. The fetus was delivered by lower segment cesarean section (LSCS) after careful separation of the membranes and avoiding damage to the vessels as there was velamentous insertion of cord with the lower margin of the placenta in the lower segment. The baby was cared for in the neonatal intensive care unit due to prematurity and discharged after six days. This case report highlights the importance of prenatal ultrasound in diagnosing vasa previa and planning an elective cesarean section with caution intraoperatively for the safe delivery of the baby.

## Introduction

The term “vasa previa,” as the name suggests, is a condition when unprotected fetal blood vessels run through the amniotic membranes and traverse the cervix below the presenting part [1,2]. The incidence may vary from one in 1,275 to one in 5,000 [2,3]. The vessels run in the amniotic membranes, without the support of the placenta and the Wharton jelly. Two types of vasa previa are reported. Type 1 (up to 90%) is associated with velamentous cord insertion. Type 2 is found in association with a bilobed placenta or a placenta with a succenturiate lobe. It may be diagnosed during early labor by vaginal examination, detecting the pulsating fetal vessels inside the internal os, or by the presence of dark-red vaginal bleeding [4,5]. Fetal mortality is at least 60% despite urgent cesarean delivery [4,5]. However, improved survival rates of over 95% have been reported with antenatal ultrasound diagnosis followed by planned cesarean section (CS). A combination of both transabdominal and transvaginal color Doppler imaging (CDI) ultrasonography (USG) provides the best diagnostic accuracy for vasa previa according to the Royal College of Obstetricians and Gynaecologists (RCOG) (2018) [4-6]. Here, we report a case of vasa previa diagnosed prenatally by ultrasound with a favorable outcome.

## Case presentation

A 29-year-old G3A2 with recurrent pregnancy loss and type 2 diabetes mellitus, conceived spontaneously after eight years of secondary infertility, at her 31st week of gestation, was admitted through emergency services in view of bleeding per vaginum (PV) with low-lying placenta. She gave a history of soakage of approximately two pads. She was clinically stable, maintaining vitals within normal range, and there was no active bleeding. Per abdomen, the uterus was of 28 weeks size, relaxed, and non-tender. The fetal heart rate was in the range of 130-140 beats/minute. She was diagnosed with placenta previa type 2 with intrauterine growth restriction and was managed conservatively with bed rest and antioxidants (L-arginine and proanthocyanidins). She was put on insulin therapy to control blood sugar. Fetal monitoring was done by daily kick count and alternate day non-stress test (NST) and weekly USG and Doppler.

**Figure 1:**
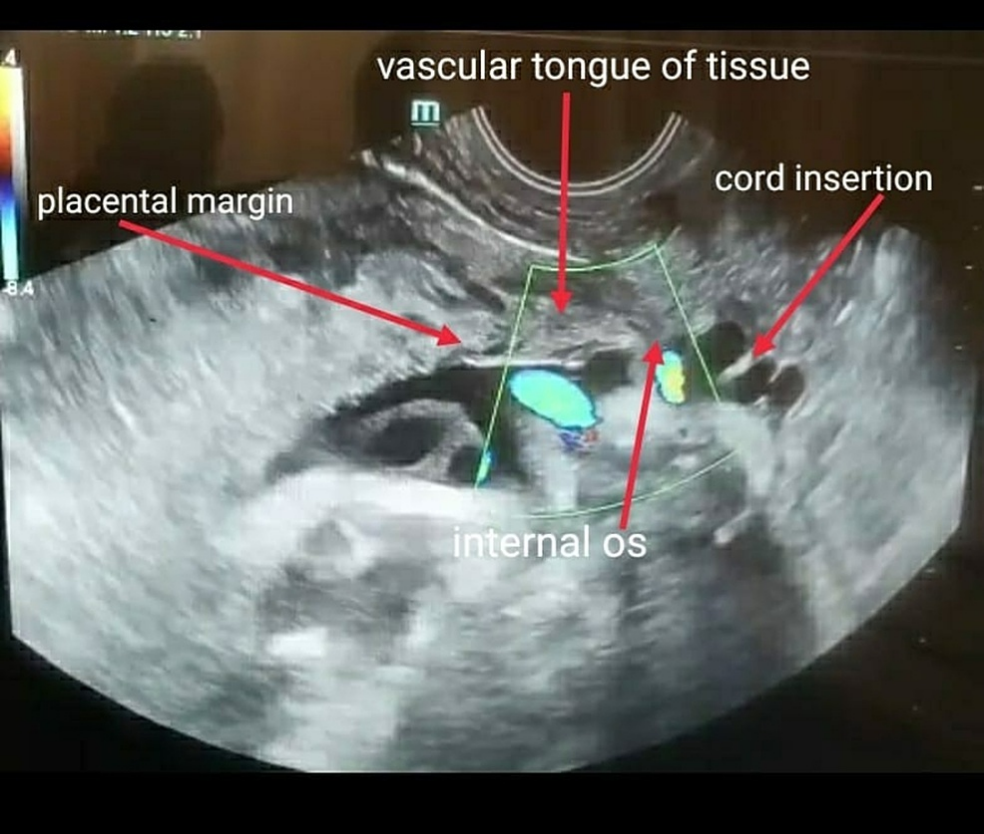
Prenatal ultrasound showing vasa previa.

Follow-up ultrasound examination at 33 weeks revealed a singleton fetus weighing 1.1 kg with a low-lying placenta, lower margin, 3 cm away from the internal os with a tongue of tissue almost reaching the internal os. Fetal blood vessels were seen running across the cervix (Figure 1).

**Figure 2:**
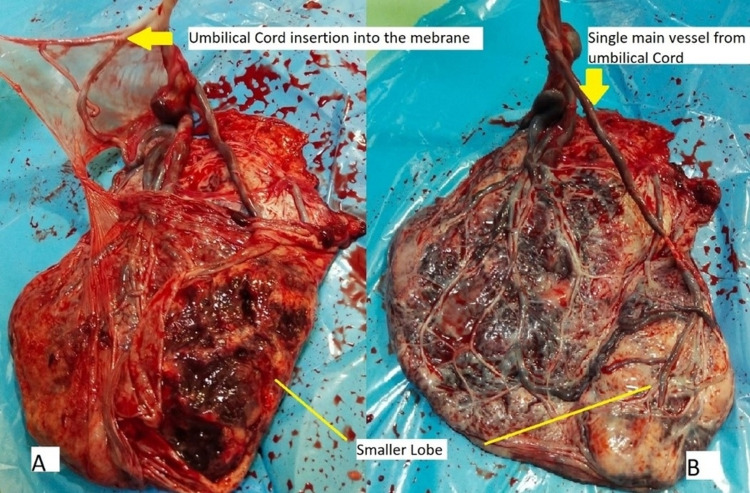
Placental examination revealed (A) umbilical cord insertion into the membrane and (B) the umbilical cord vessel and smaller lobe of the placenta.

The amniotic fluid index was 3.5 cm. The cord insertion was away from the placenta. Vasa previa was diagnosed, and early delivery by elective cesarean section or emergency cesarean section as and when required was decided if there is fetal distress or active bleeding PV. She received four doses (intramuscular) of injection dexamethasone 6 mg 12 hourly for fetal lung maturity at admission. For diabetes, she was on metformin 500 mg twice daily for seven years, which was stopped as she had intrauterine growth restriction (IUGR). She was started on injection insulin mixtard (70/30) 20 units in the morning and 15 units at night with regular blood sugar monitoring. Oral and intravenous hydration was given in view of severe oligohydramnios. Urine culture showed *Escherichia coli (E. coli)* sensitive to injection amikacin. So, an injection amikacin 375 mg intravenous twice daily was given for five days. On the second day after the diagnosis of vasa previa, the baseline fetal heart rate was 120 beats/minute, with beat-to-beat variability of 6 and three spontaneous decelerations in a 20-minute duration on NST. The patient was put on continuous electronic fetal monitoring, magnesium sulfate for neuroprotection was started, and a cesarean section was planned if there is recurrence of deceleration. She was kept under close monitoring. Elective CS was done at 34+4 weeks under spinal anesthesia. A transverse incision was made in the lower uterine segment. The fetus was delivered after careful separation of the membranes and avoiding injury to the vessels as velamentous cord insertion at the lower margin of the placenta in the lower segment was noted. A 1.55 kg male baby was delivered with an APGAR of 8/10 at one minute. There was no postpartum hemorrhage (PPH). Placental examination revealed umbilical cord insertion into the membranes and to a smaller lobe of the placenta (Figure 2A, 2B).

The baby was shifted to the neonatal intensive care unit for observation. On the fifth day, the baby was shifted to the mother’s side. Both the mother and the baby were discharged on the seventh postoperative day with the advice to follow up.

## Discussion

The reason why obstetricians should be more cautious about vasa previa is that it may be fatal for the fetus because, during contrac­tions, the presenting part may compress the fe­tal blood vessels and cause thrombosis or, due to fetal mem­brane rupture, there could be fetal hypoxia, brady­cardia, hemorrhage, and even death [1].

A hallmark symptom is painless bleeding per vaginum with dark blood and a sudden deteriora­tion of the condition of the fetus [1,4,7]. In case of bleeding from the uterus, in most cases, it is due to associated placenta previa or detachment or avulsion of the unsupported fetal vessels [1 ]. The fetus may lose a large amount of blood, and the cardioto­cogram shows a sinusoidal pattern. Fetal blood loss of 100 mL or more may cause hemorrhagic shock and death [1,8].

Ultrasound examination is a gold­ standard diagnostic test for vasa previa. In the second trimester (18-20 weeks), it is essential to determine by ultrasound the exact cord attach­ment site in all pregnant women, especially when the placenta is implanted in the lower segment. Sonographic features are usually consistent. Transabdominal color Doppler sonography demonstrates flow within vessels, which are seen overlying the internal cervical os [5]. Non-Doppler (grayscale) images may reveal echogenic parallel or circular lines within the placenta near the cervix [5]. Transvaginal ultrasound has a sensitivity of 100% and a specificity of 99%-99.8% when added with color Doppler [5,9]. In the current case, we needed to confirm using transvaginal USG with Doppler.

In such cases, ultrasound diagnosis guides us in intraoperative decision-making for a lower transverse laparotomy and a lower uterine segment uterine incision to reduce the possibility of damaging the vessels [1,10]. Similar steps were followed in our case. The membranes were separated gently, running vessels were identified, and the membranes were dissected at the vessel-free areas very cautiously, leading to a safe delivery of the fetus.

## Conclusions

Prenatal ultrasonographic diagnosis of vasa previa is of utmost importance to plan the mode of delivery to save the life of the fetus. It also contributes to extra caution in intraoperative management, which may be necessary to avoid any life-threatening complication during cesarean section in such patients. In this case, it has helped us to give a live baby to a woman with recurrent pregnancy loss with no living issues. Thus, prenatal ultrasound may be a useful tool for the evaluation of such high-risk cases before proceeding with intervention.
